# The effectiveness of patient-centred medical home model versus standard primary care in chronic disease management: protocol for a systematic review and meta-analysis of randomised and non-randomised controlled trials

**DOI:** 10.1186/s13643-018-0887-2

**Published:** 2018-11-29

**Authors:** James Rufus John, Shima Ghassempour, Federico Girosi, Evan Atlantis

**Affiliations:** 1 School of Nursing and Midwifery, University of Western Sydney, Sydney, New South Wales Australia; 2 Capital Markets Cooperative Research Centre, Level 3, CMCRC, 2/55 Harrington St, Sydney, New South Wales 2000 Australia; 3 Research Implementation Science and eHealth Group, Faculty of Health Science, University of Sydney, Sydney, New South Wales Australia; 4 Translational Health Research Institute, School of Medicine, Western Sydney University, Sydney, New South Wales Australia; 5 School of Medicine, University of Adelaide, Adelaide, South Australia Australia

**Keywords:** Patient-Centred Medical Home, Patient-focused care, Chronic disease management, Care coordination, Integrated primary care, Systematic review

## Abstract

**Background:**

Studies suggest that the Patient-Centred Medical Home (PCMH) model of primary care is more effective than standard care for improving clinical outcomes in patients with chronic diseases (non-communicable diseases), but the strength of the evidence base is unclear. The aim of the proposed systematic review is to generate a current synthesis of relevant studies on the effectiveness of PCMH model of primary care versus standard care in chronic disease management.

**Methods:**

Electronic databases such as MEDLINE, CINAHL, Embase, Cochrane Library, and Scopus will be searched using predefined search terms for PCMH, primary care, and chronic diseases for articles published up to November 2018. Reference lists of included articles and relevant reviews will also be hand searched. This review will consider eligible randomised controlled trials and controlled trials against predetermined criteria including two or more principles of PCMH model endorsed by Australian Medical Association. Data extraction will be performed independently by two reviewers, and retrieved papers will be assessed for quality using JBI Critical Appraisal Tools. Where possible, quantitative data will be pooled in statistical meta-analysis using the R packages ‘Meta’ and ‘metafor’. Effect sizes will be expressed as odds ratio (for categorical data) and weighted mean differences (for continuous data) and their 95% confidence intervals will be calculated for meta-analysis; robustness will be explored using sensitivity analysis. Heterogeneity will be assessed narratively and statistically using the Q statistics and visualised using Baujat plots including subgroup or sensitivity analyses techniques where possible. Where statistical pooling is not possible, the findings will be presented narratively.

**Discussion:**

The findings of the proposed systematic review will provide the highest level of evidence to date on the effectiveness of the PCMH model versus standard primary care in chronic disease management. We believe that our findings will inform patients, primary care providers, and public health administrators and policy-makers on the benefits and risks of PCMH model of care.

**Systematic review registration:**

PROSPERO CRD42018085378

**Electronic supplementary material:**

The online version of this article (10.1186/s13643-018-0887-2) contains supplementary material, which is available to authorized users.

## Background

The health burden from non-communicable diseases including major depressive disorder, diabetes, stroke, ischaemic heart disease, chronic obstructive pulmonary disease (COPD), lung cancer, and musculoskeletal disorders has risen in recent decades [1]. Consequently, the growing burden of multiple chronic medical conditions (multimorbidity) presents significant challenges on health systems worldwide and emphasises the need to explore better strategies towards chronic disease management [2, 3]. Patients with multimorbidity have complex health care needs that are challenging to manage in primary care for a range of reasons [4, 5]. These patients often experience poor health-rated quality of life, physical, and mental health, as well as increased risk of mortality [6, 7]. Furthermore, multimorbidity is associated with an increased risk of hospitalisations and inappropriate polypharmacy [8, 9]. Interventions and strategies that improve the quality and performance of general practice could lead to better patient outcomes and yield significant reductions in avoidable health care utilisation and overall health care costs [4].

Health care systems in high-income countries typically focus on the ‘single-disease framework’, where the delivery of primary care for the management of multimorbidity is often fragmented, lacking integration, and continuity of care [10]. Paradoxically, primary care is ideally placed to facilitate coordinated and continuous care in the management and possibly prevention of chronic diseases [11]. Strategies for effective management and prevention of multimorbidity should include integrated, multidisciplinary team (MDT), and long-term chronic disease approaches to adequately address the complex care needs of these patients [12].

The patient-centred medical home (PCMH) care model, initially introduced as “medical home” by the American Academy of Paediatrics in 1967, has been considered to conceptually provide highest quality of primary care for patients with multimorbidity [13]. Although definitions vary and there have been calls for changes [14, 15], the PCMH model typically includes a general practitioner (GP) and MDT working together to provide coordinated and patient-centred care that promotes long-term patient engagement using a long-term chronic disease approach [15, 16]. There is a small but growing body of evidence including a systematic review published in 2013 [13] suggesting that the PCMH primary care model is more effective than standard care for improving clinical outcomes in patients [17, 18], quality of care [19, 20], and reducing hospital admissions [21, 22]. Although the previous review [13] provided a comprehensive synthesis of relevant studies on the effectiveness of PCMH model of care at the time, the authors included studies in patients with multimorbidity only, thereby studies in patients with single chronic disease were excluded and studies in non-primary care settings (tertiary care services) reporting results favouring the effectiveness of the PCMH [23, 24] which may have partially biased their overall positive results and conclusions. Further, additional studies evaluating the effectiveness of the PCMH care model have been published since [25–27].

Guidelines released by the Royal Australian College of General Practitioners (RACGP) include a vision of equitable access to high-quality health care based on the PCMH [16]. Similarly, the Australian Medical Association (AMA) recognises the potential of the PCMH model of care to enable an integrated MDT approach for the management of multimorbidity [15]. Since the advocacy and implementation of the PCMH model of primary care are rapidly growing in Australia and worldwide [12, 13, 28], a current review is timely and warranted. Our searching of electronic databases and registries (PROSPERO, CDSR, JBI database for systematic reviews, and DARE) in December 2017 confirmed the absence of any newly completed or ongoing relevant systematic reviews. Therefore, the proposed systematic review described in this study protocol will aim to summarise the best available evidence on the effectiveness of PCMH models of primary care compared to standard care in chronic disease management.

## Methods

### Research design and methodology

This protocol was developed with guidance from the Centre for Reviews and Dissemination’s (CRD) Guidance for undertaking reviews in health care and Preferred Reporting Items for Systematic review and Meta-Analysis Protocols (PRISMA-P) statement [29, 30] (Additional file 1). This systematic review protocol has been registered with PROSPERO, the International Prospective Register of Systematic Reviews hosted by the Centre for Reviews and Dissemination (registration #CRD42018085378).

### Eligibility criteria

We selected specific inclusion and exclusion criteria using the Population, Interventions, Comparators, Outcomes, and Study designs (PICOS) framework (summarised in Table 1) [31].

**Table 1:** Summary of PICOS components

➢ Participants—primary care patients aged at least 18 years with one or more chronic disease ➢ Intervention—AMA-recognised PCMH principles (must meet 1 and 2 criteria) 1) Integrated or MDT care AND 2) One or more of the following principles: i. Coordination of care ii. Data driven quality of care iii. Long-term patient-provider relationship iv. Patient empowerment and patient engagement ➢ Comparison—standard primary care ➢ Outcomes i. Patient outcomes ii. Hospital outcomes iii. Economic outcomes ➢ Study design—randomised controlled trials	

#### Types of participants

This review will consider adult populations (over 18 years of age) treated for one or more chronic disease in a primary care setting. We will use the Australian Institute of Health and Welfare (AIHW) definition of chronic diseases as a wide group of conditions, illnesses, and diseases that are characterised by long-lasting and persistent effects leading to potentially intense and severe health ramifications [32]. Although chronic diseases comprise a diverse group of physical conditions, the commonly reported conditions including arthritis, asthma, back pain, cancer, cardiovascular diseases (CVD), COPD, diabetes, kidney diseases, and mental disorders.

#### Types of interventions

This review will include studies which satisfy the following criteria based on the principles of PCMH recommended by the AMA [15, 33]:

1. Integrated primary health care or MDT approaches

GP led integrated care or MDT approaches consisting of at least one other health care professional (i.e. specialists, practice nurses, and other allied health care professionals), AND

2) Any one or more other following principles of PCMH

i. *Coordination of care*
  Coordination of services within levels of the health care system (e.g. secondary/tertiary, allied health, and community services) using efficient referral pathways between sites of care.
ii. *Data driven quality of care*
  Use of technology in the development and implementation of care plans, shared decision-making, and for quality improvement auditing.
iii. *Long-term patient-provider relationship*
  Promotion of continuity of care through an ongoing partnership between patient, GP, and the MDT health care professionals towards the shared goal of providing high-quality patient-centred care.
iv. *Patient empowerment and patient engagement*
  Education to empower patients to actively participate in the development and self-management of their chronic disease. Patient values, preferences, and autonomy should always be considered in these.

#### Types of comparisons or control groups

Eligible studies must include a standard care comparison group by any definition.

#### Types of outcomes

This review will consider studies that report clinically relevant outcomes which include, but are not limited to hospital outcomes (e.g. service use, admissions, and emergency department visits), patient clinical outcomes (e.g. medical, psychological, and physical/functional), and economic outcomes (e.g. health care costs, cost-effectiveness, and cost-benefit analyses).

#### Types of studies

This review will consider RCTs and controlled trials using any alternate method of treatment group allocation. If adequate information from controlled trials is not available, cohort studies with a control group will be considered for inclusion.

### Exclusion criteria

We will exclude studies where there is genuine uncertainty regarding meeting any of the eligibility criteria (e.g. the PCMH model), duplicate publications, and papers published in any language other than English. Reasons for exclusion of studies will be reported as supplementary information.

### Search strategy

A comprehensive literature search will be performed to identify published articles up to November 2018 through searching of electronic databases such as MEDLINE, CINAHL, Embase, Cochrane Library, and Scopus. A three-step search strategy will be undertaken in this review. Records retrieved from the electronic databases will be downloaded to Endnote X8 reference manager and screened independently by two researchers. Full text copies of potentially relevant articles will be reviewed against the eligibility criteria for inclusion. A flowchart of the selection process will be produced following the PRISMA guidelines [29]. An initial pilot search of MEDLINE will be used to identify text words and phrases in the title and abstract used to index relevant articles. A sample search strategy of MEDLINE is presented in Additional file 2. A second search will be undertaken subsequently using identified index terms and keywords across all included databases by two reviewers independently. Finally, bibliographies of included studies and key review articles will be hand searched to identify any relevant studies that were missed.

### Data extraction

Data extraction will be performed independently by two reviewers using Excel spreadsheet software (Microsoft Excel, Microsoft Corporation) [34]. Extracted data will include important characteristics of the studies included for review (Additional file 3). At least two attempts will be made to contact authors for missing information or data queries. Discrepancies will be resolved through discussion to achieve consensus with a third author.

### Quality assessment

The methodological validity of the articles included for the review will be appraised independently by two reviewers using JBI Critical Appraisal Tools [35]. Discrepancies or disagreements at any stage (i.e. search strategy, data extraction, and quality assessment) will be resolved through discussion with a third author to achieve consensus.

### Strategy for data synthesis

A quantitative synthesis, where possible, is planned to pool data from included studies. Results will be subjected to double data entry to ensure data quality and reduce the possibility of error. The results will be pooled using a random-effects meta-analysis with standardised mean differences for continuous data and odds ratios for binary data and their 95% confidence intervals will be calculated. Heterogeneity will be assessed narratively and statistically using the Q statistics and visualised using Baujat plots, including subgroup or sensitivity analyses techniques where possible. We will also explore subgroup analyses based on the different study designs or characteristics included in this review where possible. Where statistical pooling is not possible, the findings will be presented narratively. We will grade the body of evidence for recommendations following the approach proposed by the Grading of Recommendations, Assessment, Development and Evaluation (GRADE) Working Group [36].

## Discussion

The systematic review outlined in this protocol paper aims to identify, assess, and synthesise the best available evidence on the effectiveness of the PCMH model of primary care for chronic diseases management. We anticipate that the proposed systematic review will be the most comprehensive evidence summary to date and believe that the findings of our systematic review will provide the most conclusive evidence (high quality) [36] on the effectiveness of PCMH care model in chronic disease management. We expect that our findings will inform patients, primary care providers, and public health administrators and policy-makers on the benefits and risks of the PCMH model in chronic disease management in primary care.

The proposed review will provide reliable information on the benefits of the PCMH model such as improving quality of care, clinical outcomes, and reducing hospital and ED admissions [17, 19, 21]. This knowledge could potentially empower patients to take proactive action and work with their GP to better self-manage their chronic conditions [37]. Empowered patients are more likely to seek better quality of care from their GPs or shop for GPs providing PCMH care, where they can be partners and not just bystanders in making decisions about their health care management [38].

We anticipate that our findings will be useful to GPs and primary care providers also, which may lead to transformational organisational changes in their structures and practices. This may encourage primary care services to be efficient in better targeting of health services according to the needs of the local community [39]. Providers will also be informed about current practices that have adopted the core PCMH principles in providing coordinated care through MDT approaches which have resulted in improved job satisfaction, burnout rates, and patient-provider relationship [40, 41].

Finally, the proposed systematic review findings might help policy-makers and health ministries in understanding health and economic benefits associated with the PCMH model of care [42, 43]. This will help direct scarce health care resources towards improvements in general practice service delivery. In addition, we also believe that the findings of this review will impact current practice, policy, and implementation guidelines [28, 44], which might result in efficiency gains in health systems in Australia and possibly other similar countries.

In summary, the proposed systematic review aims to address existing knowledge gaps by providing the highest level of evidence on the effectiveness of PCMH in chronic disease management. We expect that our findings will better inform patients, primary care providers, and policy makers on the benefits and risks associated with the PCMH model of care. In addition, the proposed systematic review may provide perspectives in achieving efficiency gains in health systems and future research opportunities.

## Additional files

Additional file 1: PRISMA-P 2015 Checklist. (DOCX 34 kb)

Additional file 2: Sample search strategy using MEDLINE. (DOCX 17 kb)

Additional file 3: Data extraction form for experimental studies. (XLSX 10 kb)

## Abbreviations

AIHW: Australian Institute of Health and Welfare
AMA: Australian Medical Association
CDSR: Cochrane Database of Systematic Review
CI: Confidence intervals
CINAHL: Cumulative Index to Nursing and Allied Health Literature
COPD: Chronic obstructive pulmonary disease
CRD: Centre for Reviews and Dissemination
CVD: Cardiovascular disease
DARE: Database of Abstract of Reviews of Effects
ED: Emergency department
GP: General practitioner
GRADE: Grading of Recommendations, Assessment, Development and Evaluation
JBI: Joanna Briggs Institute
MDT: Multidisciplinary team
PCMH: Patient-Centred Medical Home
PICOS: Population, Interventions, Comparators, Outcomes, and Study designs
PRISMA: Preferred Reporting Items for Systematic review and Meta-Analysis
PRISMA-P: Preferred Reporting Items for Systematic review and Meta-Analysis Protocols
RCT: Randomised controlled trials
RR: Risk ratio
