# Supplementary material for: The effectiveness of patient-centred medical home model versus standard primary care in chronic disease management: protocol for a systematic review and meta-analysis of randomised and non-randomised controlled trials
Source: Syst Rev. 2018 Nov 29;7:215. doi: 10.1186/s13643-018-0887-2 (PMC6267917; doi:10.1186/s13643-018-0887-2)
Supplement: Supplementary file 2 — Sample search strategy using MEDLINE. (DOCX 17 kb) [file 13643_2018_887_MOESM2_ESM.docx]

**Sample search strategy using MEDLINE**

| **No** | **Search terms** |
| --- | --- |
| 1 | PCMH.tw. |
| 2 | (patient-centred adj medical adj home*).tw. |
| 3 | (patient adj centred adj medical adj home*).tw. |
| 4 | (patient-centered adj medical adj home*).tw. |
| 5 | (patient adj centered adj medical adj home*).tw. |
| 6 | (Medical adj home*).tw. |
| 7 | (Home adj based adj care).tw. |
| 8 | (home adj based adj model).tw. |
| 9 | (Health adj home*).tw. |
| 10 | (Health adj care adj home*).tw. |
| 11 | (Health-care adj home*).tw. |
| 12 | (Patient adj centred adj care).tw. |
| 13 | (Patient-centred adj care).tw. |
| 14 | (Patient adj centered adj care).tw. |
| 15 | (Patient-centered adj care).tw. |
| 16 | (Patient adj focused adj care).tw. |
| 17 | (Patient-focused adj care).tw. |
| 18 | (Integrated adj primary adj care).tw. |
| 19 | (Integrated adj care).tw. |
| 20 | (Integrated adj health adj care).tw. |
| 21 | (Integrated adj service*).tw. |
| 22 | (Integrated adj delivery).tw. |
| 23 | (Team-based adj care).tw. |
| 24 | (multidisciplinary adj care*).tw. |
| 25 | (care adj team).tw. |
| 26 | (care adj coordination).tw. |
| 27 | (coordinated adj care).tw. |
| 28 | (coordinated adj health adj care).tw. |
| 29 | (coordinated adj primary adj care).tw. |
| 30 | (collaborative adj practice).tw. |
| 31 | (Collaborative adj care).tw. |
| 32 | (Advanced adj primary adj care).tw. |
| 33 | (enhanced adj primary adj care).tw. |
| 34 | (augmented adj care).tw. |
| 35 | (augmented adj service*).tw. |
| 36 | (guided adj care).tw. |
| 37 | (chronic adj care adj model*).tw. |
| 38 | (Patient adj aligned adj care adj team).tw. |
| 39 | (patient adj care adj team).tw. |
| 40 | 1 or 2 or 3 or 4 or 5 or 6 or 7 or 8 or 9 or 10 or 11 or 12 or 13 or 14 or 15 or 16 or 17 or 18 or 19 or 20 or 21or 22 or 23 or 24 or 25 or 26 or 27 or 28 or 29 or 30 or 31 or 32 or 33 or 34 or 35 or 36 or 37 or 38 or 39 |
| 41 | (primary adj health adj care).tw. |
| 42 | (family adj practice*).tw. |
| 43 | (primary adj care*).tw. |
| 44 | (community adj network*).tw. |
| 45 | (health adj care adj coalitions).tw. |
| 46 | (chronic adj care*).tw. |
| 47 | (primary adj physician*).tw. |
| 48 | (primary adj care adj physician*).tw. |
| 49 | (general adj practice*).tw. |
| 50 | (general adj physician*).tw. |
| 51 | (general adj practitioner*).tw. |
| 52 | (community adj based adj provider*).tw. |
| 53 | (community adj practice).tw. |
| 54 | (community adj care).tw. |
| 55 | (preventive adj service*).tw. |
| 56 | (patient adj care).tw. |
| 57 | Adult*.tw. |
| 58 | (middle adj age*).tw. |
| 59 | geriatric.tw. |
| 60 | (geriatric adj practice).tw. |
| 61 | elder*.tw. |
| 62 | exp Chronic Disease/ |
| 63 | (Chronic adj disease*).tw. |
| 64 | (Chronic adj illness*).tw. |
| 65 | exp COMORBIDITY/ |
| 66 | comorbid*.tw. |
| 67 | multimorbid*.tw. |
| 68 | exp Diabetes Mellitus/ |
| 69 | ((Diabetes adj mellitus) or Diabet*).tw. |
| 70 | exp ASTHMA/ |
| 71 | Asthma*.tw. |
| 72 | exp ARTHRITIS/ |
| 73 | Arthritis.tw. |
| 74 | exp Back Pain/ |
| 75 | (Back adj pain).tw. |
| 76 | exp Cardiovascular Diseases/ |
| 77 | (cardiovascular adj disease*).tw. |
| 78 | (Heart adj disease*).tw. |
| 79 | exp Neoplasms/ |
| 80 | cancer*.tw. |
| 81 | (malignant adj neoplasm*).tw. |
| 82 | exp Pulmonary Disease, Chronic Obstructive/ |
| 83 | (chronic adj obstructive adj pulmonary adj disease).tw. |
| 84 | (respiratory adj disease*).tw. |
| 85 | exp Kidney Diseases/ |
| 86 | (Kidney adj disease*).tw. |
| 87 | 41 or 42 or 43 or 44 or 45 or 46 or 47 or 48 or 49 or 50 or 51 or 52 or 53 or 54 or 55 or 56 or 57 or 58 or 59 or 60 or 61 or 62 or 63 or 64 or 65 or 66 or 67 or 68 or 69 or 70 or 71 or 72 or 73 or 74 or 75 or 76 or 77 or 78 or 79 or80 or 81 or 82 or 83 or 84 or 85 or 86 |
| 88 | 40 and 87 |
| 89 | Randomized Controlled Trials as Topic/ |
| 90 | (Randomized adj controlled adj trial*).tw. |
| 91 | (Randomised adj controlled adj trial*).tw. |
| 92 | (Clinical adj Trial*).tw. |
| 93 | Random adj allocat* |
| 94 | (Clinical adj trial).pt. |
| 95 | (Controlled adj trial*).tw. |
| 96 | 89 or 90 or 91 or 92 or 93 or 94 or 95 |
| 97 | 88 and 96 |
| 98 | limit 97 to (English language and humans) |
